# UV-Induced Mutants of *Metarhizium anisopliae*: Improved Biological Parameters, Resistance to Stressful Factors, and Comparative Transcriptomic Analysis

**DOI:** 10.3390/jof11060412

**Published:** 2025-05-27

**Authors:** Hao Gao, Yingjie Luo, Qiming Li, Jiaxuan Guo, Bin Wang

**Affiliations:** 1 Anhui Provincial Key Laboratory of Biological Control, Anhui Agricultural University, Hefei 230036, China; gh3195152315@163.com (H.G.); yingjieluo010434@163.com (Y.L.); qimingli@stu.ahau.edu.cn (Q.L.); 18648221135@163.com (J.G.); 2 Key Laboratory of Pine Wood Nematode Disease Prevention and Control, State Forestry and Grassland Administration, Hefei 230031, China

**Keywords:** entomopathogenic fungi, UV-irradiation, environmental stress, virulence, transcriptome

## Abstract

*Metarhizium anisopliae*, a well-known species of entomopathogenic fungi with great potential as a biological control agent, is vulnerable to UV damage, which restricts its use in the field. To improve the fungal resistance to UV irradiation, UV-induced mutant strains of *M. anisopliae* s. l. were screened and compared with the wild-type (WT) strain for heat resistance, growth rate, conidial yield, and virulence. Comparative transcriptomic analysis between the selected UV-resistant mutants and the WT was carried out. The results showed that the five mutants exhibited significantly higher heat resistance and growth rates, while the conidial production remained unchanged. Among them, the mutant MaUV-22 exhibited enhanced tolerance to heat, oxidative, osmotic, and SDS stresses as well as increased virulence against *Galleria mellonella*. Moreover, the transcriptome analysis of MaUV-22 revealed that the expression of genes associated with the heat shock protein pathway, glutathione S-transferase, and thioredoxin reductase were increased dramatically, while the expression of genes related to the catalase and superoxide dismutase pathways was downregulated. The UV-induction technique is an effective strategy to improve fungal resistance to environmental stresses and affords some other beneficial traits such as better control efficacy of entomopathogenic fungi against pests in the field.

## 1. Introduction

Insect pests often pose a detrimental impact on plant growth and cause huge economic losses to crop production. Chemical control has been regarded as an efficient method against various pests. Meanwhile, the continuous and improper use of chemical pesticides leads to serious ‘3R’ problems (resistance, residue, and resurgence) [1]. Entomopathogenic fungus (EPF) is regarded as one of the most efficient alternatives to chemical insecticides by directly penetrating the insect integument and consuming the host’s nutrients and producing toxins [2,3,4]. EPF exhibits excellent traits, such as a low risk of resistance development, primarily due to the evolutionary ’arms race’ between the hosts and pathogens [5]. Moreover, EPF is safe for humans and the environment, and offers potential for sustainable pest management [6]. Therefore, some fungal pesticides have been found to be efficient to control agricultural pests [7,8]. For instance, after spraying a ULV oil formulation of *Metarhizium acridum* on nearly 6000 hectares of grasslands, the nymphal mortality of *Locusta migratoria manilensis* exceeded 90% within 11–15 days [9]. Application of a *Metarhizium flavoviride* oil-based formulation against *Locustana pardalina* (predominantly fifth-instar nymphs) in South Africa resulted in 98% nymphal mortality three weeks after treatment [10].

Although EPF has many advantages as a biological control agent, its sensitivity to abiotic stresses, including solar ultraviolet radiation (UV) and high temperatures, limits its field applications [11]. Studies have revealed that abiotic stresses severely affect conidial germination and fungal infection processes, leading to a sharp decrease in the control efficiency of target pests [12]. Meanwhile, the abiotic stress tolerance varies greatly among fungal strains [13]. Studies have demonstrated that this variation correlates with the strains’ geographical origins [14,15,16] and culture conditions [17] (culture medium [18,19,20], pH [21], and illumination [22]). As tolerance is genetically determined, physical, chemical mutagenesis, and gene editing can enhance fungal resistance [23,24,25,26]. Specifically, UV mutagenesis effectively generates mutants with improved stress tolerance and fitness traits. For instance, UV-induced *Beauveria bassiana* mutants exhibited higher growth rates, stress resistance, virulence, and enzyme activities compared with wild-type strains [27].

In this study, we employed UV mutagenesis to obtain *Metarhizium anisopliae* sensu lato mutants with enhanced abiotic stress resistance. The mutant phenotypes, including colony morphology, growth rate, conidial yield, stress tolerance, and fungal virulence, were systematically characterized against wild-type strains, while transcriptomic analysis was also conducted to reveal the underlying mechanisms.

## 2. Materials and Methods

### 2.1. Entomopathogenic Fungi and Insects

The used EPF strain *M*. *anisopliae* s. l. Ma83 (RCEF5580) is stored in the RCEF strain Bank (WDCM No. 1031), Anhui Provincial Key Laboratory of Biological Control, Anhui Agricultural University. The experimental insect *Galleria mellonella* is a lab-reared strain originally purchased from Tianjin Huiyude Biotechnology Co. Ltd. (Tianjin, China). Larvae were maintained at 25 °C and 60% relative humidity (RH) under a 12:12 h (light:dark) photoperiod until reaching the third-instar stage.

### 2.2. Monospore Strain Isolation

The conidial suspension of the Ma83 strain was adjusted to 2 × 10^2^ conidia mL^−1^, and 5 μL of conidial suspension was added to each grid area (1 cm × 1 cm) of water agar medium (agar powder 15 g, distilled water 1000 mL) marked at the bottom of the Petri dish. The grid with one conidium was confirmed by an inverted microscope (Olympus IX70, Olympus Corporation, Tokyo, Japan) and marked. A single spore on the grid-marked water agar was transferred to a new PDA plate for monospore culture and incubated at 25 °C in the dark for 10 days. The monosprore culture was stored in 20% glycerol solution (*v*/*v*) at −80 °C, and used in the study to guarantee genetic consistency.

### 2.3. Conidial Production and Conidial Suspension

The monospore culture of the Ma83 strain was grown on potato dextrose yeast agar (PDA) medium for 10 days at 25 °C in the dark. Conidia were harvested by scraping conidia from the agar surface with a sterile inoculation shovel and adding them to 100 mL sterilized 0.05% Tween-80 (*v*/*v*) with continuous magnetic stirring. The conidial suspension was filtered through a single layer of muslin cloth to remove the hyphae and debris. The conidial concentration was determined using a hemocytometer under a light microscope (Olympus model BX100, Olympus Corporation, Tokyo, Japan), and the final conidial suspensions were adjusted to the desired concentrations using 0.05% Tween 80.

### 2.4. UV-Induced Fungal Mutants

The conidial suspension of the monospore strain Ma83 was adjusted to 2 × 10^3^ conidia mL^−1^. An aliquot of 100 μL of the suspension was inoculated on the PDA medium and evenly spread over the PDA medium plates. The PDA medium plates were treated under UV-B irradiation (λ = 311 nm, distance = 20 cm, irradiation power = 31.54 W/m^2^, chamber temperature = 25 °C) by a UV-irradiation testing machine (YouKe Tech, Hefei, China), and the sample plates were periodically taken away every 15 min. Control groups were set without UV irradiation. All PDA plates from the control and treatment groups were cultured in the dark at 25 °C for 3 days for colony counting. The optimal irradiation time for fungal mutations was determined to lead to a conidial mortality of approximately 90%, which was taken as the mutation time [28].

After the optimal irradiation, each fungal colony as a potential UV-induced mutant was transferred to the center of a new PDA plate and marked with a unique code. Conidia of the UV-induced mutants and the wild-type strain (WT) were collected, and conidia suspensions were prepared at a concentration of 2 × 10^6^ conidia mL^−1^. Then, 20 μL of each suspension was inoculated separately on GB medium (5 g/L peptone, 20 g/L glucose, and 15 g/L agar) plates. The GB plates were irradiated with UV-B for 60 min and then incubated in the dark at 25 °C for 24 h. Conidial germination of over 200 conidia on each plate was examined under a microscope. The mutants with significantly high UV resistance were selected for further testing. Three biological replicates were performed for each treatment.

$$Germination\;rate\;(\%)=\frac{Germinated\;conidia}{Total\;conidia}\times 100\%$$

$$Relative\;germination\;rate\;(\%)=\frac{Germination\;rate\;of\;treatment}{Germination\;rate\;of\;control}\times 100\%$$

### 2.5. Fungal Growth and Conidial Productivity of UV-Induced Mutants

An aliquot of 2 μL of conidia suspension of 2 × 10^6^ conidia mL^−1^ for each UV-resistant mutant and the WT strain was added to the center of the PDA medium and then cultured at 25 °C in the dark. Colony diameters were measured every other day for 14 days to assess the growth rates of the fungal mutants. To assess conidial productivity, three pieces of mycelial mat were sampled at the middle of the radius of the mutant colony with a puncher (∅6 mm) and transferred into a centrifuge tube containing 5 mL of 0.05% Tween-80. After mixing well for 10 min on a shaker, the concentration of the conidial suspension was determined using a hemocytometer, and the conidial amount per cm^2^ was calculated. Three biological replicates were performed for each treatment.

### 2.6. The Tolerance of UV Mutant to Environmental Stresses

One milliliter of 2 × 10^6^ conidia mL^−1^ conidial suspension for each UV-resistant mutant was added to a new 1.5 mL Eppendorf tube, and the Eppendorf tube was treated separately in a thermal bath at 45 °C for 0.5 h and 1 h, respectively. After the heat shock, 20 μL of the conidial suspension was transferred to GB medium and cultured at 25 °C in the dark for 24 h. The conidial germination rate of each mutant was determined under a microscope.

Resistance of the UV-induced mutant to oxidation or osmotic stress was tested by adding each of 0.5 μL/mL H_2_O_2_ (oxidative stress), 0.5 mol/L NaCl (osmotic stress), and 400 μg/mL SDS (cell wall interference stress) to the GB medium and PDA medium, respectively. An aliquot of 20 μL of conidial suspension at a concentration of 2 × 10^6^ conidia mL^−1^ was dropped and spread on the GB medium and then cultured in the dark at 25 °C for 24 h to 48 h. Conidial germination was observed under a microscope. Meanwhile, 2 μL of 2 × 10^6^ conidia mL^−1^ conidial suspension was inoculated in the center of the PDA medium with different additive concentrations and pure PDA medium (CK). The colony diameters and sporulation of the fungal colonies under different oxidation and osmotic stress were measured after culture for 10 days in darkness at 25 °C. Fungal tolerance to oxidation and osmotic stresses was evaluated by comparing inhibition rates between the stress-treated and the control groups of the UV-induced mutants and WT strain. Three independent biological replicates were performed for each stress condition.

$$Inhibitory\;rates\;(\%)=\frac{\left(Control-Treatment\right)}{Control}\times 100\%$$

### 2.7. Bioassay

The UV-induced MaUV-22 mutants and the WT strain were grown on a PDA medium for 10 days at 25 °C. Conidia were harvested and transferred to 100 mL sterile 0.05% Tween-80 (*v*/*v*) in flasks with continuous magnetic stirring. The conidial suspension was filtered through a single layer of muslin cloth to remove debris and mycelia, and the conidial concentration was adjusted to 1 × 10^7^ conidia mL^−1^ using a hemocytometer under a light microscope (Olympus model BX100). Thirty third-instar larvae of *G. mellonella* for each replication were immersed in the conidial suspension for 10 s and then transferred to a piece of sterile filter paper to erase the overloaded conidial suspension on the integument of *G. mellonella* larvae. The controls were mock-infected with 0.05% Tween-80. The larvae were transferred to glass Petri dishes with wet cotton to maintain high humidity and cultured at 25 °C. The larvae in each treatment were checked daily. The dead larvae were immediately transferred to aseptic Petri dishes for culturing to confirm fungal infection. All experiments were performed with three biological replicates. The data were adjusted using Abbott’s formula [29], and the time–mortality curve was plotted. The median lethal time (LT_50_) was estimated through Probit analysis in IBM SPSS Statistics (Version 22.0) and served as the virulence index (with lower values indicating higher virulence).

### 2.8. RNA Isolation, Library Preparation, and RNA Sequencing

Conidia of the WT strain (Ma83) and the MaUV-22 mutants were cultured according to Section 2.3, washed with 0.05% Tween-80 solution, and filtered through a single-layer muslin cloth to remove the mycelium and medium fragments. The conidia were collected by centrifugation at 12,000 rpm for 3 min, and the total RNA was extracted from both strains using TRIzol reagent (Invitrogen, Carlsbad CA, USA) according to the manufacturer’s instructions. RNA purity and quantification were determined by a NanoDrop 2000 spectrophotometer (Thermo Scientific, Waltham, MA, USA). RNA integrity was assessed with an Agilent 2100 Bioanalyzer (Agilent Technologies, Santa Clara, CA, USA). mRNA was further extracted from the total RNA using polyT-oligo-attached magnetic beads. Libraries were constructed using the VAHTS Universal V6 RNA-seq Library Prep Kit (Vazyme, Nanjing, China) for Illumina according to the manufacturer’s instructions. RNA samples were sequenced using the Illumina NovaSeq 6000 platform (Illumina, San Diego, CA, USA). Paired-end reads (150 bp) were then generated. Five biological replicates of each strain were subjected to RNA sequencing at OE Biotech Co. Ltd. (Shanghai, China). The raw sequence data have been submitted to the NCBI Short Read Archive (SRA) with accession number PRJNA1183655.

Quality control and adapter trimming of raw paired-end reads were performed by fastp [30] with default parameters. The clean reads were mapped to the reference genome using HISAT2 [31] (v 2.1.0). FPKM [32] of each gene was calculated and the read counts of each gene were obtained by HTSeq-count (v 0.11.2). PCA analysis was performed using R (v 3.2.0) to evaluate the biological duplication of samples. Differential expression analysis was performed using DESeq2 [33] (v 1.22.2). Adjusted *p*-value < 0.05 (Benjamini–Hochberg method) and |log_2_FC| ≥ 1 were set as the threshold for significantly differentially expressed genes (DEGs). Based on the hypergeometric distribution, GO [34] and KEGG [35] pathway enrichment analyses of the DEGs were performed to screen the significantly enriched terms using R (v 3.2.0), respectively. R (v 3.2.0) was used to draw the column diagram and bubble diagram of the significant enrichment term. Heatmap analysis of the DEGs was performed using R (v 3.2.0) to demonstrate the expression pattern of genes in different groups and samples.

### 2.9. Quantitative PCR Verification

Total RNA extraction followed the protocol described in Section 2.8. Single-strand cDNA was synthesized from 1 μg of total RNA using the HiScript III All-in-one RT SuperMix Perfect for qPCR (Nanjing Vazyme Biotech Co. Ltd., Nanjing, China) (80 °C 5 s, 40 °C 15 min). Real-time qPCR was performed on a CFX96 real-time PCR detection system (Bio-Rad, Hercules, CA, USA) with AceQTM qPCR SYBR Green Master Mix (Vazyme, Nanjing, China). The qPCR condition was: 95 °C 5 min, 40 cycles of 95 °C 10 s, and 60 °C 30 s. GAPDH was used as the reference gene. Gene expression levels were calculated by the 2^−ΔΔCT^ method. The qPCR primers are listed in Supplementary Table S1. All experiments were performed with three biological replicates.

### 2.10. Statistical Analysis

All experimental data were analyzed using IBM SPSS Statistics (Version 22.0). The normality and homogeneity of variances were assessed using the Shapiro–Wilk test and Levene’s test, respectively. For comparisons among more than two means, one-way ANOVA was performed followed by Duncan’s multiple comparison test (*p* < 0.05). Comparisons between two means were conducted using an independent samples *t*-test.

## 3. Results

### 3.1. UV-Induced Mutants

**Figure 1:**
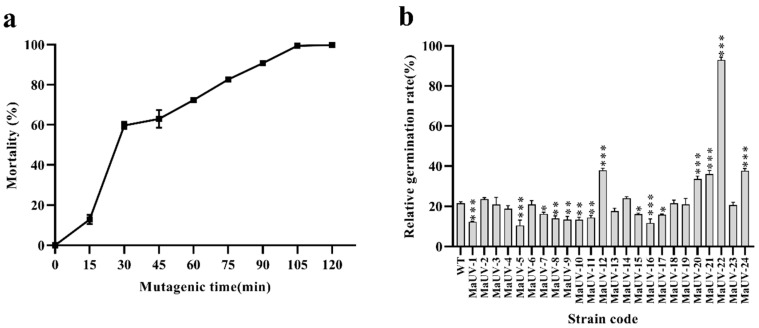
UV-induced mutants of the monospore culture of the Ma83 strain. (**a**) Dynamic tracking of conidial mortality of the Ma83 strain under UV irradiation. (**b**) Conidial germination rates of 24 mutants and the wild -type strain. In (**b**), one-way ANOVA was performed using Duncan’s multiple range test. ‘*’ *p* < 0.05; ‘**’ *p* < 0.01; ‘***’ *p* < 0.001.

Conidial mortality of the monospore culture of the Ma83 WT strain increased with longer UV irradiation time. The conidial mortality rose sharply within the first 30 min of UV exposure, and reached 100% after 120 min. Previous studies have demonstrated that 90% conidial mortality promotes forward mutation in fungal strains [28]. Therefore, the optimal irradiation time for obtaining fungal mutants was 90 min, which led to a conidial mortality of 90.7% (Figure 1a).

Twenty-four UV-induced mutants of the monospore culture of Ma-83 were isolated after UV-irradiation for 90 min and named as MaUV-1 to MaUV-24, respectively. Five of them (MaUV-12, MaUV-20, MaUV-21, MaUV-22, and MaUV-24) exhibited significantly higher UV resistance than the WT strain (Figure 1b). The MaUV-22 mutant presented the highest germination rate of 92.9% under UV irradiation for 1 h. The five selected mutants were subjected to further biological tests.

### 3.2. Fungal Growth, Conidial Productivity, and Resistance to Environmental Stresses of the UV-Induced Mutants

**Figure 2:**
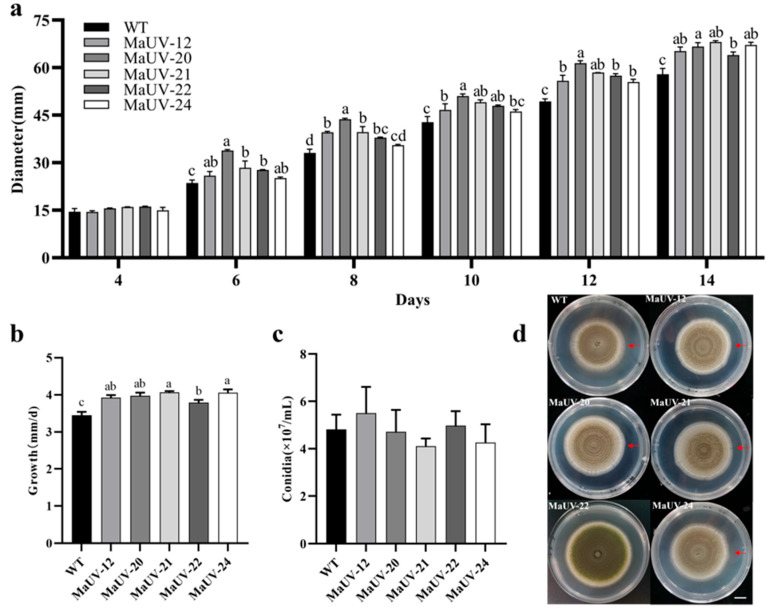
Growth rate and conidial production of the mutants and wild-type strain (WT). (**a**) Diameters of fungal colony of the mutants and the WT strain on PDA. (**b**) The average daily growth of the mutants and the WT strain on PDA in terms of fungal colony diameter. (**c**) Conidial production of the mutants and the WT strain on PDA. (**d**) Top-view images of the wild-type (WT, Ma83) and five UV-induced mutants grown on PDA medium for 14 days. The scale bar in (**d**) represents 1 cm, and the red arrow marks the colony’s maximum growth margin. Data represent the mean ± SE (*n* = 3). One-way ANOVA was performed using Duncan’s multiple range test. Different lowercase letters indicate a significant difference at *p* < 0.05.

No difference in growth rate was observed between the five mutants and the wild strain in the initial 4 culturing days. Gradually, the mutants showed higher growth rates over the WT strain, especially after a period of 12 days (Figure 2a). The average daily growth of the mutants was calculated to be significantly higher than the WT (Figure 2b). However, there was no significant difference in conidial production between the five mutants and WT strain (Figure 2c).

**Figure 3:**
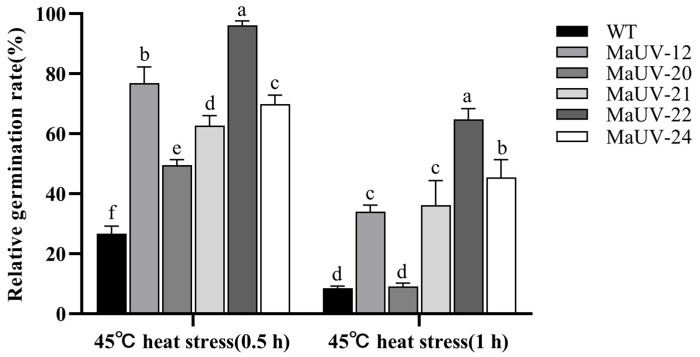
Conidial germination rate of the mutants and the WT strain under heat stress. Data represent the mean ± SE (*n* = 3). One-way ANOVA was performed using Duncan’s multiple range test. Different lowercase letters indicate significant differences at *p* < 0.05.

After suffering heat stress at 45 °C for 0.5 h, the conidial germination rates of the five mutants were significantly higher than that of the WT (26.7%), and the germination rate of MaUV-22 was the highest (96.1%, Figure 3). When the heat stress time reached 1 h, the germination rates of all strains decreased, while the mutants except for MaUV-20 still showed a higher heat resistance to the WT strain. The mutant MaUV-22 exhibited the highest germination rate of 64.8%, while the germination rate of the WT strain was decreased to 8.6% (Figure 3). Thus, the mutants exhibited a higher heat resistance than the WT strain, and MaUV-22 with the highest heat resistance was selected for further tests.

**Figure 4:**
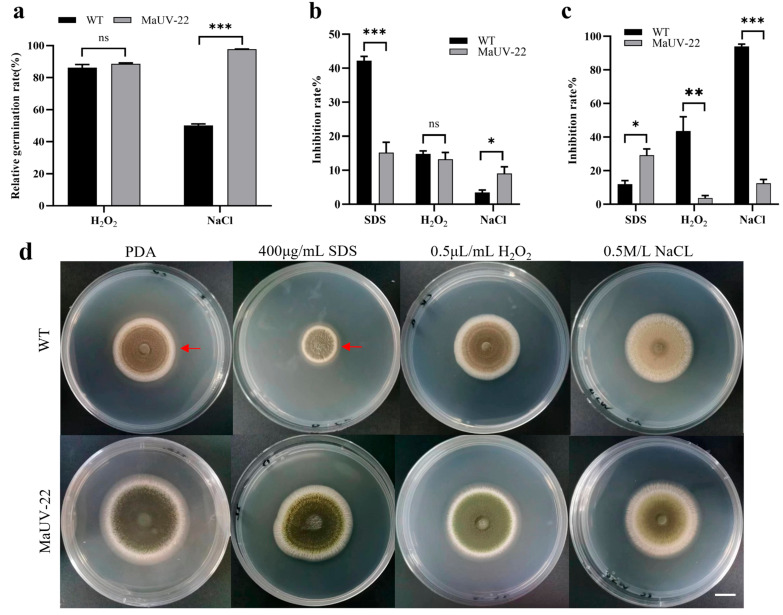
Response of the mutant MaUV-22 and WT strain to different environmental stresses on different developmental stages of conidial germination, mycelial growth, and sporulation. (**a**) Effects of three stress agents on conidial germination of the mutant MaUV-22 and the WT strain. (**b**) Relative inhibition rates of the three stress agents on the fungal colony growth of the mutant MaUV-22 and the WT strain. (**c**) Relative inhibition rates on the conidial production of the mutant MaUV-22 and the WT strain. (**d**) Top-view images of the strain (WT and MaUV-22) grown on PDA supplemented with SDS, H_2_O_2_, and NaCl for 10 days. The scale bar in (**d**) represents 1 cm, and the red arrow marks the colony’s maximum growth margin. Data represent the mean ± SE (*n* = 3). Significant differences were determined by the independent-samples *t*-tests for pairwise comparisons; ‘ns’ *p >* 0.05; ‘*’ *p* < 0.05; ‘**’ *p* < 0.01; ‘***’ *p* < 0.001.

The mutant MaUV-22 and WT strain showed different responses to various stresses, including cell membrane stress of 400 μg/mL SDS, oxidative stress of 0.5 μL/mL H_2_O_2_, and osmotic stress of 0.5 mol/L NaCl, at their different developmental stages such as conidia germination, mycelium growth, and sporulation. In the conidial germination stage, the mutant MaUV-22 and the WT strain were inhibited by SDS, but was slightly affected by H_2_O_2_. The mutant MaUV-22 showed a significantly higher resistance to the NaCl stress than the WT strain, with a germination rate of 97.68% for the mutant and 50.20% for the WT strain (Figure 4a). During the mycelium growth stage, the mutant MaUV-22 had a higher resistance to the cell membrane stress, lower resistance to the osmotic stress, and similar resistance to the oxidative stress compared with the WT strain (Figure 4b). In the sporulation stage, SDS reduced the sporulation of the mutant MaUV-22 by 29.2%, while the WT strain showed a reduction of 12.00%. On the contrary, the stresses of H_2_O_2_ and NaCl led to a sharp decrease in sporulation of the WT strain by a reduction of 43.6% and 93.8%, respectively, while the sporulation of the mutant MaUV-22 was reduced by only 3.75% and 12.55%, respectively (Figure 4c).

### 3.3. Fungal Bioassays

**Figure 5:**
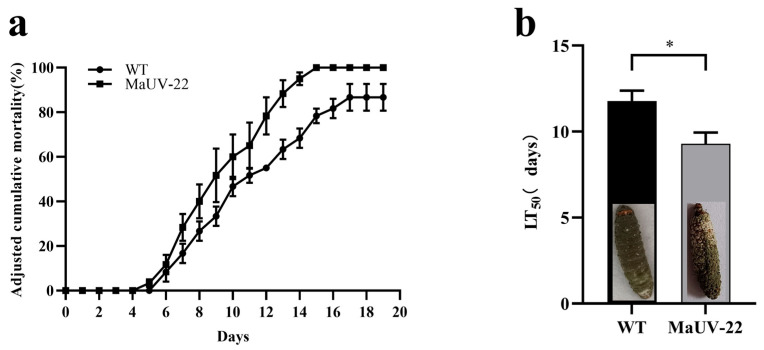
Virulence of the mutant MaUV-22 and the WT strain against the larvae of *G. mellonella*. (**a**) The cumulative mortality of *G. mellonella* larvae inoculated with the conidial suspension at a concentration of 1 × 10^7^ conidia mL^−1^. The controls were mock-infected with 0.05% Tween-80. The adjusted cumulative mortality was recorded from 0 to 19 d. Data represent Abbott’s corrected mortality values. (**b**) Median lethal time (LT_50_) of MaUV-22 and WT. Data represent the mean ± SE (*n* = 3). Significant differences were determined by the independent-samples *t*-test for pairwise comparisons. ‘*’ *p* < 0.05.

The bioassay revealed that the mutant MaUV-22 exhibited a higher virulence against *G. mellonella* larvae than the WT strain. The mutant MaUV-22 led to a 100% mortality of the larvae, while the WT strain only led to a mortality of 76.67% on the 15th day of bioassay (Figure 5a). Compared with the WT strain, the mutant MaUV-22 showed a significantly faster knocking down speed to *G. mellonella* larvae with an LT_50_ of 9.18 d, 3.02 d less than that of the WT strain (Figure 5b), indicating the enhanced virulence of the mutant MaUV-22.

### 3.4. RNA-Sequencing and DEG Analyses

#### 3.4.1. RNA-Sequencing

**Figure 6:**
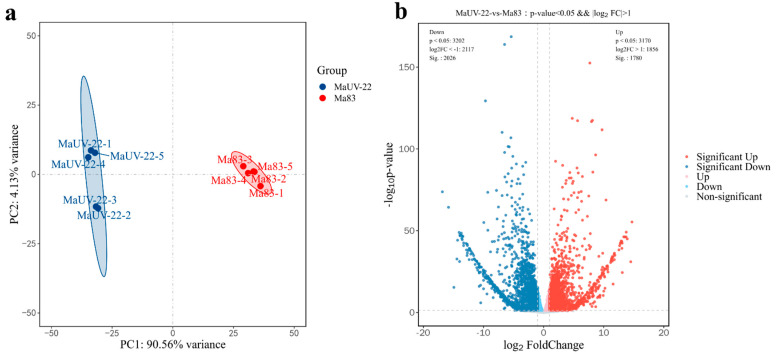
MaUV-22 versus WT (Ma83) RNA sequencing analysis. (**a**) Principal component analysis (PCA). Each point represents a sample, and similar samples are close to each other. The confidence is the solid line range of the ellipse. (**b**) DEGs volcanic plots of MaUV-22 versus WT (Ma83). Each dot represents a gene, where the blue dot represents downregulated genes while the red dot represents upregulated genes.

After filtering for high-quality sequences, 44.8 and 41 M clean reads were obtained from the wild-type (WT, Ma83) and mutant strain (MaUV-22), respectively. The clean reads were mapped to the reference genome sequence (RefSeq: GCA_013305495.1), and the mean comparison ratio was 77.89%. All Q30 values (sequencing quality > 99.9%) were over 95%, indicating the reliability of the data (Table S2). In the principal component analysis (PCA), there was a separated distribution between the WT and MaUV-22 samples, representing a significant difference between the two groups (Figure 6a). A total of 3806 differentially expressed genes (DEGs) were detected in MaUV-22 versus WT (*p*-value < 0.05 and |log_2_FC| > 1.0) including 1780 upregulated genes and 2026 downregulated genes (Figure 6b).

Functional annotation of the DEGs revealed significant differences in gene expression between the mutant MaUV-22 and the WT strain. The upregulated genes mainly included putative subtilisin, stress-responsive A/B barrel domain protein, dynamin family protein, putative ribonucleoprotein, transposase, partial argininosuccinate synthase, methyltransferase type 11, and many hypothetical proteins (Table S3). The downregulated genes mainly included the putative agmatine deiminase, cell wall protein, methionine aminopeptidase, serine/threonine-protein kinase Sgk2, putative ferric-chelate reductase, putative agmatine deiminase and partial catalase, methyltransferase, DNA helicase PIF1, nitrate reductase, C6 finger domain-containing protein, Peptidase M24, structural domain protein, Formate/nitrite transporter, Heat shock protein DnaJ, and some hypothetical proteins (Table S4).

#### 3.4.2. Enrichment Analysis of DEGs

**Figure 7:**
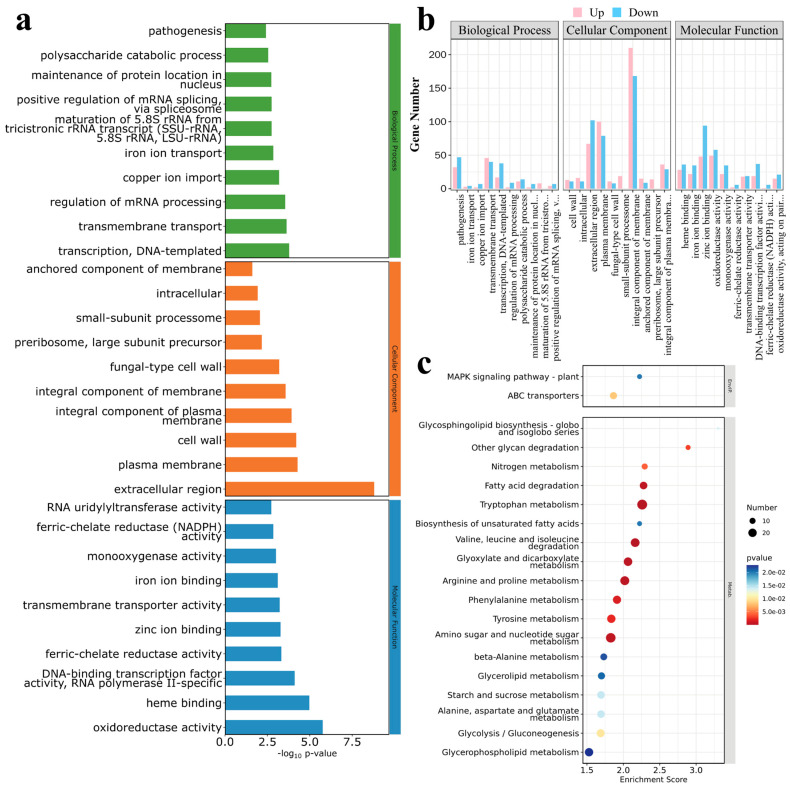
DEGs enrichment analysis of MaUV-22 versus WT. (**a**,**b**) Gene Ontology (GO) analysis of the DEGs, and the DEGs were divided into three groups including biological processes, cellular components and molecular functions. In (**a**), the Vertical coordinate shows the GO term names, and the horizontal coordinate presents −log_10_*p*-value; in (**b**), the Vertical coordinate indicates the gene count, and the horizontal coordinate presents GO terms names. Specifically, the complete names of the abbreviated GO terms on the horizontal axis are as follows: maintenance of protein location in nucleus; maturation of 5.8S rRNA from tricistronic rRNA transcript (SSU-rRNA, 5.8S rRNA, LSU-rRNA); positive regulation of mRNA splicing, via spliceosome (BP); integral component of plasma membrane (CC); DNA-binding transcription factor activity, RNA polymerase II-specific; ferric-chelate reductase (NADPH) activity; and oxidoreductase activity, acting on paired donors, with incorporation or reduction of molecular oxygen (MF). (**c**) Kyoto Encyclopedia of Genes and Genomes (KEGG) analysis of DEGs. Vertical coordinate shows the name of the KEGG metabolic pathway, and the horizontal coordinate presents the enrichment scores of genes annotated to the pathway.

GO and KEGG analyses of the DEGs indicated that the DEGs between the mutant MaUV-22 and the WT strain were assigned to 94 functional groups belonging to three GO terms: biological processes (bp), cellular components (cc), and molecular functions (mf). The DEGs in the biological processes were mainly related to the function of DNA-templated transcription, transporting, mRNA regulation, and pathogenesis, etc. Concerning the DEGs in the cellular components, extracellular and intracellular regions, plasma membrane, cell wall, etc. were mainly affected. In the molecular functions, the DEGs were found in oxidoreductase activity, heme binding, DNA-binding transcription factor activity, RNA polymerase II-specific, and ion bonding, etc. (Figure 7a). Statistically, more upregulated genes appeared in the cell components, but more downregulated genes appeared in the biological processes and molecular functions (Figure 7b).

A total of 30 pathways, including cell processing, environmental information processing, genetic information processing, metabolism, and organism systems, were enriched by KEGG analysis. Among them, DEGs in the metabolism pathway played a dominant role, and the top 20 pathways with the highest DEG contents are shown in Figure 7c. In the metabolism pathway, six carbohydrate metabolism, seven amino acid metabolism/degradation, two lipid metabolism, and one organic acid metabolism were enriched. In addition, glycosphingolipid biosynthesis and ABC transporters were also significantly enriched, which may have played a key role in fungal resistance to environmental stresses.

#### 3.4.3. Quantitative PCR Validation of DEGs

**Figure 8:**
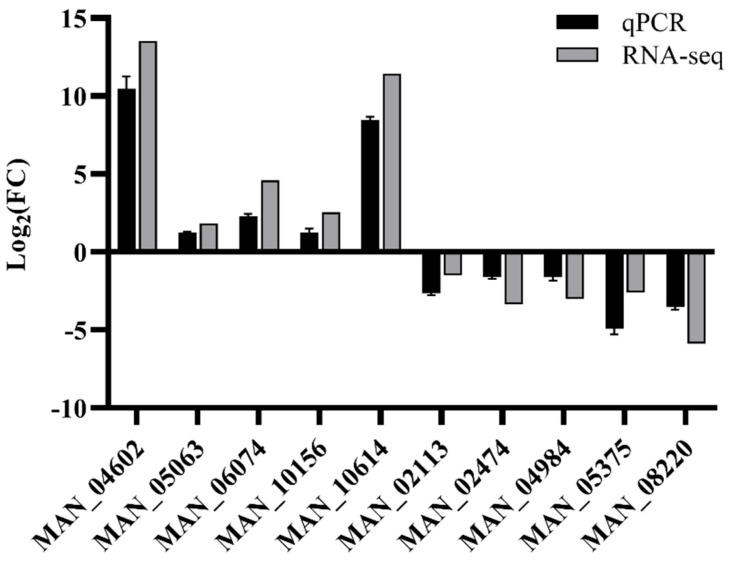
Validation of the gene expression in the transcriptome by qPCR. The qPCR and RNA-Seq data represent the log_2_ fold-change (FC) values of selected genes, calculated by the ΔΔCt method and DESeq2, respectively. The qPCR data are presented as the mean ± standard error (SE) of three biological replicates.

Transcriptome sequencing was validated by randomly selecting five upregulated and five downregulated DEGs for qRT-PCR confirmation. The selected 10 genes showed consistent up- or downregulated expression between the qPCR and transcriptome analyses, indicating the reliability of the transcriptome sequencing (Figure 8).

#### 3.4.4. Expression Levels of Stress-Resistant and Virulence-Related Genes

Transcriptomic analysis revealed different expression levels of stress resistance and virulence-related genes between the MaUV-22 and WT strain. Heatmap analysis revealed that two putative trehalose-6-phosphate synthase/trehalose phosphatase (*MAN_05271* and *MAN_06118*) and one ortholog of trehalose phosphate synthase (*MAN_05293*) were significantly upregulated in MaUV-22. Furthermore, two α-1,2-mannosyltransferase orthologs (*Alg11*: *MAN_01420* and *Ktr4*: *MAN_04041*) showed similar upregulated patterns (Figure S1). Notably, the majority of DEGs encoded heat shock proteins (HSPs), including *Hsp30* (*MAN_02994* and *MAN_10156*), *Hsp70* (*MAN_00546*, *MAN_05778*, *MAN_05779*, and *MAN_09859*), *Hsp90* (*MAN_09612*), and *Hsp98* (*MAN_02202)*, which were upregulated in MaUV-22 (Figure S2). The enhanced stress resistance of the MaUV-22 strain may be related to the synergistic upregulation of these genes.

The heatmap analysis of antioxidant enzyme genes revealed that multiple catalase orthologous genes (*MAN_00866*, *MAN_05231*, *MAN_06166*, and *MAN_09251*) and two superoxide dismutase orthologous genes (*MAN_04984* and *MAN_06660*) were significantly downregulated in the MaUV-22 strain (Figure S3a). However, it is worth noting that multiple glutathione S-transferase orthologous genes (*MAN_00700*, *MAN_01297*, *MAN_04181*, *MAN_06177*, *MAN_09595*, and *MAN_10370*) and two thioredoxin reductase genes (*MAN_01312* and *MAN_05758*) were upregulated significantly in the MaUV-22 strain (Figure S3b,c). These results indicate that the maintenance of cell redox balance may be mainly regulated by glutathione S-transferase and thioredoxin reductase in the MaUV-22 strain.

Subtilisin-like proteases (*Pr1*) and chitinases are regarded as essential determinants of the virulence of entomopathogenic fungi. Unexpectedly, transcriptome analysis showed that most subtilisin-like protease genes in the MaUV-22 strain were significantly downregulated (Figure S4a). However, most chitinase genes in this strain were upregulated significantly (Figure S4b). We hypothesize that the upregulated chitinase genes in MaUV-22 may enhance fungal penetration through the insect cuticle, potentially compensating for the virulence attenuation caused by downregulated *Pr1* expression.

## 4. Discussion

Microbial breeding is an efficient pathway to improve the genetic bases of microbes. The technique process includes chemical mutagens, physical mutagens, and genetic engineering [26,27,36,37]. Chemical mutagens such as 4-nitroquinoline-1-oxide [38] and *N*-methyl-*N’*-nitro-*N*-nitrosoguanidine [39] may pose some health and ecological risks. Although genetic engineering breeding is effective, it is uncertain whether it can be commercialized due to the regulation of genetic transgenic technology [40,41,42]. Therefore, physical mutagenesis is often viewed as the first choice for microbial breeding. In physical mutagenesis, ultraviolet mutagenesis is reported to be a safe, simple, and low-cost operation, as demonstrated in *Beauveria bassiana* [27], *Metarrhizium anisopliae* [43], and *Halomonas smyrnensis* [44].

In this study, the UV-induced mutant MaUV-22 exhibited significant superiority to the WT strain in resistance to oxidation, osmotic, SDS, and heat stresses, consistent with previous research [27,43]. Moreover, MaUV-22 showed significantly higher virulence than the WT strain. High environmental resistance and virulence are valuable features for entomopathogenic fungi in the field control of pests [11,43], since entomopathogenic fungi rely on conidia for host infection [45]. During the infection process, fungal pathogens must endure nutrient limitation, osmotic stress, and oxidative bursts within the host [46]. The combination of high virulence and robust environmental stress resistance renders MaUV-22 a promising candidate for pest control applications. The mutant MaUV-22 also showed significant improvement in the fungal growth rate, but no significant difference in conidial production was observed between the five mutants and WT strains that revealed an inconsistency between sporulation and other biological traits. Previous studies also verified that the sporulation of UV-induced fungal mutants may be increased or decreased [27,43], indicating that sporulation is a trade-off of fungal traits.

Transcriptome analysis showed that most of the upregulated genes in GO enrichment were related to the cell wall and plasma membrane. The cell wall and plasma membrane of fungi not only help maintain cell structure and vitality, but also regulate permeability, resist osmotic pressure and mechanical stress, thus play a key role in stress response and signal transduction [47,48]. When cells are under stress, the cell wall integrity pathway (CWI) is activated and interacts with the Hog1-MAPK pathway to synergistically regulate fungal stress responses [49]. Notably, membrane processes depend on energy and metabolic support. The KEGG enrichment analysis revealed that most DEGs were significantly enriched in metabolic pathways, suggesting that UV induction alters the strain’s metabolic and energy balance. Studies demonstrate that propionic acid metabolism not only mediates fungal heat stress responses via endocytosis promotion, but also generates propionyl-CoA, which enters the methyl citrate cycle [50,51]. As a key metabolic node, this cycle regulates carbon/nitrogen assimilation and hyphal growth [52] while modulating the glyoxylate cycle [53] (virulence-linked) and TCA cycle [54] (core carbon metabolism). We propose that propionic acid and glyoxylate metabolism converge at the methyl citrate cycle to coordinately enhance the MaUV-22 strain’s growth, stress adaptation, and virulence.

Transcriptome analysis for the mutant MaUV-22 and WT strain revealed that many DEGs in the study were related to virulence and stress resistance. Our findings demonstrate that the mutant MaUV-22 exhibited a significant upregulation of the trehalose biosynthesis pathways and heat shock protein (HSP) genes, along with altered expression in antioxidant enzyme systems. These transcriptional changes are likely attributed to UV mutagenesis-induced modifications in key metabolic and protective pathways, ultimately enhancing the strain’s stress tolerance and virulence. For example, trehalose in cytoplasmic solute is a typical stress metabolite, which is involved in cell resistance and regarded as a pressure-resistant inducer [24,55]. The function of highly conserved heat shock proteins has been verified to play essential roles in fungal response to stress, heat resistance, osmosis, and so on [56,57]. *Hsp30* is a hydrophobic plasma membrane protein whose expression is induced by alterations in membrane fluidity (e.g., heat stress-induced changes) [56]. Studies demonstrate that *Hsp30* expression in *Saccharomyces cerevisiae* is significantly upregulated under heat or osmotic stress conditions [58]. Unlike *Hsp30*, *Hsp70* functions as a highly conserved molecular chaperone that mediates cellular stress responses [59,60]. In the entomopathogenic fungus *B. bassiana*, *Hsp70* has been shown to play critical roles in both stress resistance and virulence regulation [61]. Both *Hsp70* and *Hsp90* are involved in MAPK signaling pathway regulation. In *S. cerevisiae*, Hsp70 modulates MAPK *Slt2* signaling through its co-chaperone *Fes1* [62], while Hsp90 interacts with Slt2 [62]. In *Candida albicans*, Hsp90 mediates crosstalk between the Hog1-MAPK and *Slt2* (*Mkc1*) pathways [63]. Our transcriptomic analysis revealed that multiple heat shock protein orthologs and key trehalose metabolic genes were significantly upregulated in the MaUV-22 strain, which likely contributed to the improved fungal tolerance to the stresses.

Antioxidant enzymes are also believed to pose their effects on fungal resistance to adverse environments, especially UV irradiation and oxidative stress [64,65,66]. Under these stress conditions, intracellular reactive oxygen species (ROS) accumulate. Under physiological steady-state conditions, ROS can participate in intracellular signal transduction [67], but excessive ROS can damage cell viability [68]. Therefore, in response to the stresses, the regulation of ROS appears to be important for the fungi. In our DEG analysis, the same results appeared in the two systems that maintain cellular redox homeostasis and intracellular ROS levels. The genes of glutathione S-transferase and thioredoxin reductase were greatly upregulated. However, genes in the catalase and superoxide dismutase pathways were downregulated. This is somewhat similar to the results of Sun [27]. We hypothesize that the synergy of the glutathione S-transferase and thioredoxin reductase systems may constitute a primary regulatory mechanism for maintaining cellular redox homeostasis in the MaUV-22 strain. However, the mechanism needs further study.

In addition, previous studies have verified that polyketide synthase helps *B. bassiana* conidia resist UV damage [69]. Chen [70] found that the zinc finger transcription factor BbCmr1 is involved in the stress resistance of *B. bassiana*. The same α-1,2-mannosyltransferase (Ktr4) is involved in the thermotolerance and UV-B resistance of *B. bassiana* [71]. A Velvet protein VeA in the central developmental pathway (CDP) of *B. bassiana* has also been reported to be involved in stress tolerance [72]. The polyketide synthase gene (*MAN_00012*), the zinc finger domain-containing protein gene (*MAN_07799*), *Ktr4*, and *VeA* genes were significantly upregulated in our study, which confirmed the resistant functions of these genes.

Moreover, fungal appressoria cells as well as clastic enzymes are closely related to cuticle degrading, hyphae penetrating, and virulence [2,3,4]. However, subtilisin-like Pr1 proteases, except for *Pr1E* and *Pr1F*, were downregulated in this study, although previous studies have shown that Pr1 proteases contribute 19–29% virulence in cuticle degradation [73]. Notably, the majority of chitinase genes displayed an inverse expression pattern. Many enzymes are involved in the fungal infection of insects. Our results confirmed that the virulence of mutant strains is determined by comprehensive synergy in related enzymes. The genes involved in the functional enzymes need to be well-studied.

Our research revealed that UV irradiation is an efficient pathway to obtain fungal mutants of *M. anisopliae* s. l., with significantly improved virulence and environmental resistance. The improved fungal traits originate from their genetic mutation induced by UV irradiation. The present study provides some clues for a better understanding of the UV-induced mutagenesis mechanism of fungi. However, it is important to acknowledge that all experiments were performed under controlled laboratory conditions, which may not fully replicate the complex biotic and abiotic interactions in natural ecosystems. Specifically, the biocontrol efficacy and ecological adaptation of the MaUV-22 strain under field conditions require further evaluations. Future studies should prioritize field trials to validate these findings.

## Data Availability

The raw data supporting the conclusions of this article will be made available by the authors upon request.

## Supplementary Materials

The following supporting information can be downloaded at: https://www.mdpi.com/article/10.3390/jof11060412/s1, Table S1. Primers used for qPCR verification. Table S2. RNA sequencing of the mutant MaUV-22 and the WT strain of *M. anisopliae*. Table S3. A list of top 20 up-regulatory genes of MaUV-22 mutant versus Ma83(WT). Table S4. A list of top 20 down-regulatory genes of MaUV-22 mutant versus Ma83(WT). Figure S1. Gene expression heatmap of trehalose and mannose. Each small square represents a gene, and its color represents the expression level of the gene. Red indicates a high expression gene, and blue indicates a low expression gene. All following representations adhere to this same color convention. Figure S2. Heatmap of heat shock protein gene expression. Figure S3. Expression heatmap of antioxidant enzyme genes. (a): Catalase and superoxide dismutase gene; (b): Glutathione reductase and glutaredoxin gene; (c): Thioredoxin reductase and thioredoxin gene. Figure S4. Expression heatmap of virulence genes. (a): Subtilisin-like protease gene; (b): Chitinase gene.
