# Supplementary material for: UV-Induced Mutants of Metarhizium anisopliae: Improved Biological Parameters, Resistance to Stressful Factors, and Comparative Transcriptomic Analysis
Source: J Fungi (Basel). 2025 May 27;11(6):412. doi: 10.3390/jof11060412 (PMC12194297; doi:10.3390/jof11060412)
Supplement: Supplementary file 1 [file jof-11-00412-s001.zip › Supplementary Materials.pdf]

Table S1. Primers used for qPCR verification

Gene	Primer sequence (5'-3')
MAN_04602	ACAGCAGGCAGAATGGCTTC<br>AACAATACCGTCCGCCGAG
MAN_05063	CAGCGAGATCGAATCCGTCA<br>GAGAGTTGGGATGACGCCAA
MAN_10156	GATCTCTCGACCTTGCCTCG<br>AAGTTTGACGTCCGCGAGAT
MAN_10614	CCCACGAGAGAAGGCAATGT<br>GAGGGTGCCGGATTTACCAA
MAN_06074	CACACTCTCTCGCCATCTCC<br>GGTACACAGCAAAAGCGACG
MAN_08220	CAGACGAGATGACGCACAGA<br>CGTCAAAGCTCGCATCACTG
MAN_04984	CCTGGCACGATGGAAGAGAG<br>AAGCAAGCAACGGTAACTGC
MAN_02474	ACGTAAGAGTGCCTCCTTGC<br>ATGGAAGCTGGATGTGAGCC
MAN_05375	GATCTCCTTGTCGTGGTGCA<br>TCTTTTCTCGTGACGTCCCG
MAN_02113	TATGTCCAGGCAGCAACAGG<br>TTGGAGTGCGTTGGTGATGA
GAPDH	GACTGCCCGCATTGAGAAG<br>AGATGGAGGAGTTGGTGTTG

Table S2. RNA sequencing of the mutant MaUV-22 and the WT strain of *M. anisopliae*

Sample	Raw Reads(M)	Clean Reads(M)	Total reads	Total mapped reads	Valid Bases(%)	Q30(%)
Ma83-1	43.51	42.13	42128198	35225906	96.83	97.26
Ma83-2	46.89	45.49	45491940	38865007	97.02	96.97
Ma83-3	43.04	41.44	41441170	33870858	96.28	97.01
Ma83-4	49.75	48.18	48175468	38645996	96.83	96.74
Ma83-5	48.49	47.1	47096050	38604380	97.12	96.94
MaUV-22-1	43.08	41.52	41515338	31601226	96.37	96.94
MaUV-22-2	41.37	40.05	40048486	26142454	96.81	96.88
MaUV-22-3	40.16	39.02	39024144	25284204	97.18	96.97
MaUV-22-4	41	39.66	39655926	30776758	96.73	96.89
MaUV-22-5	46.27	44.89	44885554	36825018	97	97

Table S3. A list of top 20 up-regulatory genes of MaUV-22 mutant versus Ma83(WT)

Gene ID	Log2 Fold Change	p-value	Annotation
MAN_10062	14.71	4.72E-56	hypothetical protein, partial
MAN_10061	14.51	1.02E-31	hypothetical protein
MAN_01861	13.92	1.03E-45	hypothetical protein
MAN_10776	13.75	7.76E-50	hypothetical protein
MAN_08782	13.72	6.32E-47	putative subtilisin
MAN_04602	13.52	6.92E-47	stress responsive A/B barrel domain protein
MAN_10746	13.33	5.76E-47	hypothetical protein
MAN_10876	13.09	3.83E-25	hypothetical protein
MAN_07756	13.07	4.16E-43	hypothetical protein
MAN_10777	13.03	6.27E-40	Dynamin family protein
MAN_10063	13.01	3.34E-44	hypothetical protein
MAN_08781	12.97	1.28E-43	hypothetical protein
MAN_10826	12.91	2.09E-44	hypothetical protein, partial
MAN_06843	12.81	2.74E-43	Methyltransferase type 11, partial
MAN_06674	12.77	6.28E-43	putative ribonucleoprotein
MAN_10613	12.66	1.44E-41	hypothetical protein, partial
MAN_07875	12.54	5.46E-39	hypothetical protein
MAN_05638	11.86	1.15E-35	argininosuccinate synthase, partial
MAN_08798	11.79	6.04E-37	hypothetical protein, partial
MAN_10843	11.77	5.09E-33	transposase

Table S4. A list of top 20 down-regulatory genes of MaUV-22 mutant versus Ma83(WT)

Gene ID	Log2 Fold Change	p-value	Annotation
MAN_09251	-16.85	1.67E-74	catalase, partial
MAN_09730	-15.83	4.81E-65	hypothetical protein
MAN_00287	-14.93	4.35E-16	methyltransferase, partial
MAN_04599	-14.43	2.00E-33	hypothetical protein
MAN_06249	-14.32	6.62E-45	putative agmatine deiminase
MAN_01670	-14.06	1.14E-49	cell wall protein
MAN_10713	-13.98	6.69E-32	DNA helicase PIF1, ATP-dependent, partial
MAN_09805	-13.92	3.24E-48	nitrate reductase, partial
MAN_09210	-13.91	1.43E-47	hypothetical protein, partial
MAN_03771	-13.70	5.91E-48	C6 finger domain-containing protein, partial
MAN_09387	-13.69	3.19E-49	methionine aminopeptidase
MAN_05716	-13.62	4.54E-46	hypothetical protein
MAN_03766	-13.54	3.07E-45	hypothetical protein, partial
MAN_09386	-13.53	1.86E-47	Peptidase M24, structural domain protein, partial
MAN_10813	-13.43	3.26E-47	serine/threonine-protein kinase Sgk2
MAN_09843	-13.32	1.30E-41	hypothetical protein
MAN_07665	-13.32	3.23E-43	Formate/nitrite transporter, partial
MAN_07019	-12.91	6.49E-43	hypothetical protein
MAN_01915	-12.83	6.06E-39	Heat shock protein DnaJ, partial
MAN_01882	-12.74	1.47E-35	putative ferric-chelate reductase

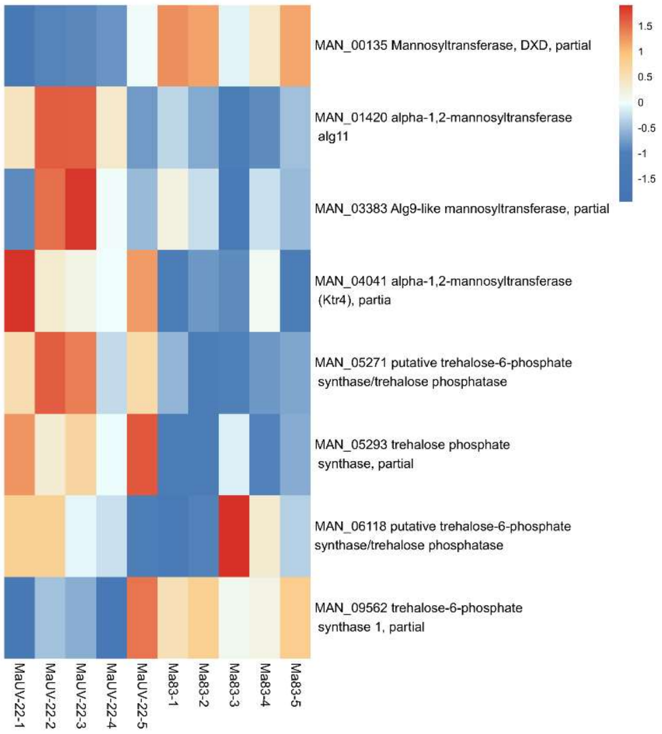

Figure S1. Gene expression heatmap of trehalose and mannose. Each small square represents a gene, and its color represents the expression level of the gene. Red indicates a high expression gene, and blue indicates a low expression gene. All following representations adhere to this same color convention.

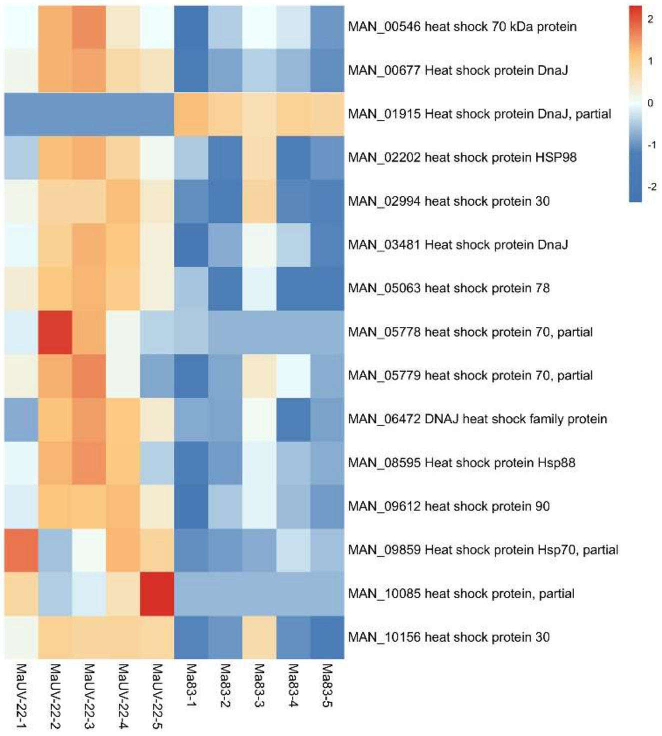

Figure S2. Heatmap of heat shock protein gene expression.

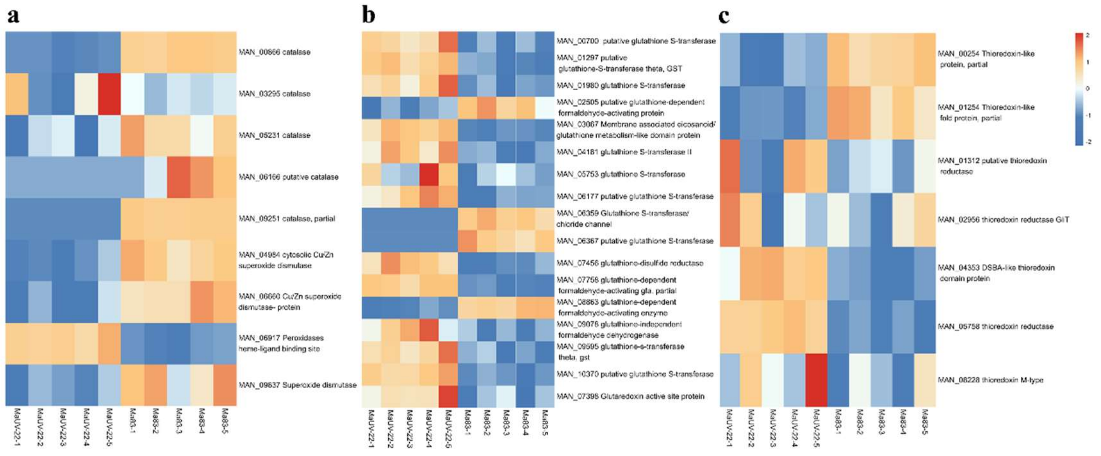

Figure S3. Expression heatmap of antioxidant enzyme genes. (a): Catalase and superoxide dismutase gene; (b): Glutathione reductase and glutaredoxin gene; c: Thioredoxin reductase and thioredoxin gene.

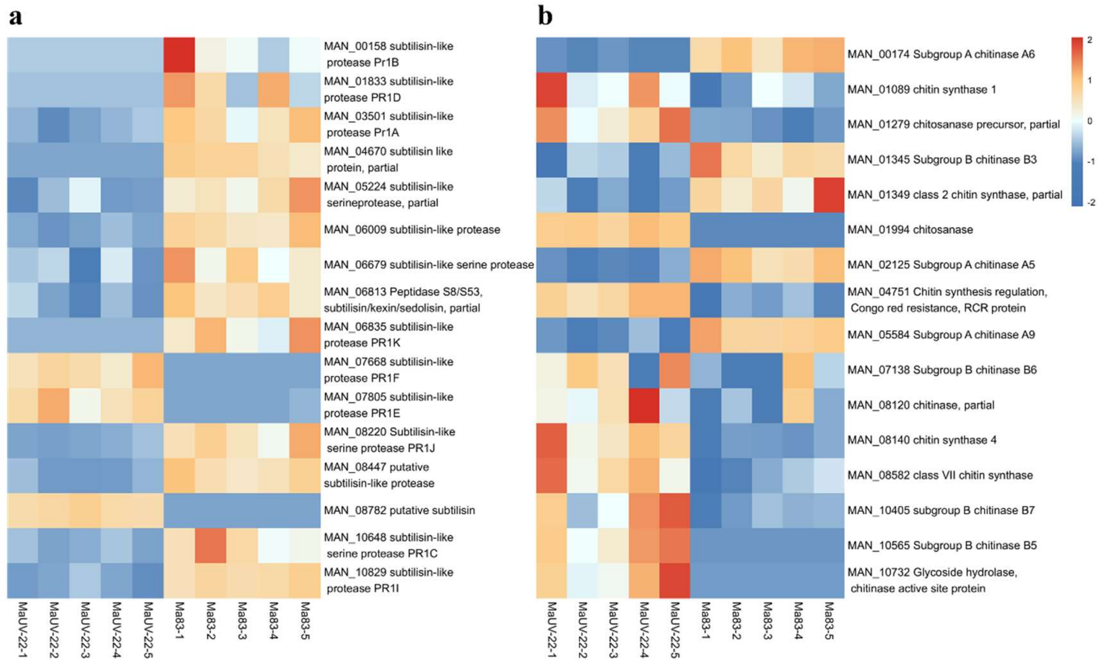

Figure S4. Expression heatmap of virulence genes. (a): Subtilisin-like protease gene; (b): Chitinase gene
